# The New Attempt at Modeling of the Three-Dimensional Geometry of the Epidermal Skin Layer and the Diffusion Processes of Nanomolecular Drug Carriers in Such Structures

**DOI:** 10.3390/molecules28010205

**Published:** 2022-12-26

**Authors:** Mariola M. Błaszczyk, Jerzy P. Sęk

**Affiliations:** Department of Chemical Engineering, Faculty of Process and Environmental Engineering, Lodz University of Technology, 213 Wólczańska St., 90-924 Lódź, Poland

**Keywords:** stratum corneum, nanoparticles, diffusion, skin, drug delivery

## Abstract

Nanoparticles are presently considered the efficient carriers of medicals, cosmetics, and pharmaceuticals in the human organism. There is a lot of research carried out on the delivery of these materials in a non-invasive way. Such a method is very safe in times of global illnesses and pandemics. The most frequently investigated route is the approach to delivering nano-media through the skin as the result of diffusion processes. The stratum corneum, the outermost layer of skin, is the most resistive barrier to such a form of penetration. In this work, a new model is proposed to predict nanoparticles’ transport through this layer. It introduces the concept of the three-dimensional model of the stratum corneum, which allows to define the skin surface area from which diffusion occurs. This structure was replaced by the single capillary, resulting from theoretical considerations. Modeling of the diffusion process of nanoparticles as the result of Brownian motion in such a capillary was performed numerically using COMSOL Multiphysics package programs. Further, using the dimensions of such a capillary, a new model of diffusion was developed in which the parameters allow to determine the effective diffusion coefficient as a function of nanoparticle size and the viscosity of a liquid. As a result, the proposed models provide a new and efficient approach to the determination of the nano-molecules’ transport phenomena through the skin layer.

## 1. Introduction

Nanosized mineral particles are commonly present in the human environment, from which they can penetrate living organisms. Such nanoparticles are considered dangerous suspensions, creating smog, and they are responsible for many illnesses. However, in the last decades, there has appeared an approach in which medicines and cosmetics can contain nanoparticles as the main active component. These particles, entering the organisms as drug carriers, can bring about medically and cosmetologically positive results. Most frequently, the nanoparticles are delivered as aerosols directly to the lungs or as suspensions placed on the skin surface, from which they diffuse into the organisms as a part of nanomedical approaches [1,2,3]. 

The skin is the human body’s biggest organ, with a surface of about two square meters and a weight of up to 10–20% of the total mass. It protects organisms against external mechanical, chemical, and biological shocks from surroundings, infections, excessive temperature, and radiation. It is also responsible for preserving the proper thermal balance of the body, the sweating processes’ rate, and perception signals from an environment [4,5]

**Figure 1:**
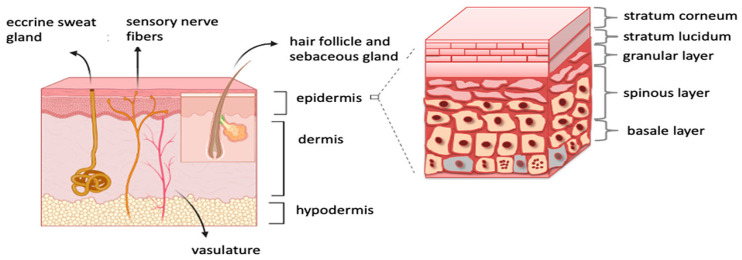
The cross-section of skin layers and the epidermis.

The human skin is a multilayered membrane with a layer named the epidermis as the outer one. The cross-section of this stratum is presented in Figure 1 [6]. 

Among these layers, the stratum corneum, with a thickness of up to twenty micrometers and built from the keratinocytes, is the most obstructive to the migration of the chemical substances, lipids, proteins, vitamins, and other substances to the interior of the body. Problems with trans epidermis diffusion become even more meaningful when the transport of nanomedical and nano-cosmetical substances is considered. These media, with solid particles the size of nanometers, would move very slowly or even be trapped inside the narrow ducts of the stratum corneum. 

The stratum corneum consists of dead keratinocytes separated by lipid layers. It usually consists of fifteen such layers, meaning the total thickness of this stratum is generally up to sixteen micrometers. Modeling approaches to the transport through the stratum corneum available in the literature [7,8,9,10,11,12,13] consider this layer as a two-dimensional vertical wall where keratinocytes are bricks and lipid layers are mortar filling the gaps. Such an approach does not allow, however, for an estimation of diffusion rates from the surface of the skin to inside the body, even when the three-dimensional model was presented the area of diffusion was not considered [11].

The research presented in this work can be a development of the present state-of-the-art. The introduction of the three-dimensional model of the stratum corneum is an improvement over the already existing approaches. It allows for considering the surface of the skin from which external substances migrate into organisms. Further, in the presented work, the mosaic of bricks fitted in the lipidic mortar was replaced by the capillary duct where the diffusion processes can occur. 

Transport of nanoparticles in such a capillary was simulated numerically using the COMSOL Multiphysics [14,15] package of programs, considering Brownian and Stokes’ forces acting on them. 

The next step of modeling was focused on the approximation of numerical results with an appropriate function. For this purpose, as a result of theoretical considerations, the new formula was built based on Fick’s first law. This allowed estimating the values of the effective diffusion coefficient of nanoparticles in the stratum corneum as the function of process parameters, such as sizes of nanoparticles and the viscosity of the liquid in which diffusion occurs. Prediction of these parameters makes it possible to calculate the diffusion rate and amount of mass transferred through the stratum corneum. 

## 2. Modeling Considerations

### 2.1. Capillary Model of Stratum Corneum

**Figure 2:**
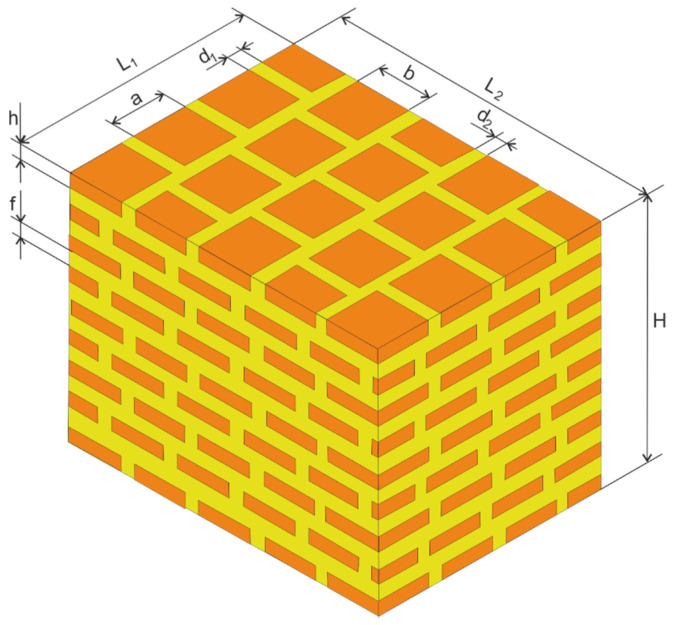
The three-dimensional model of the stratum corneum.

A three-dimensional model of the stratum corneum developed for this work is presented in Figure 2.

Its size, L_1_ · L_2_, corresponds to the area of the skin on which the process of migration of external substances into a living organism is considered. 

Other dimensions are as follows:

a and b—Dimensions of keratinocytes,

f—Thickness of the lipid layer,

h—Thickness of the layer of keratinocytes equals the height of a single keratinocyte, 

d_1_ and d_2_—Width of the lipid channels,

H—Thickness of the stratum corneum.

The surface of such an area can be expressed as:

(1) $$S=L_{1}\cdot L_{2},$$

Considering the sizes of keratinocytes and lipid channels, the dimensions L_1_ and L_2_ of the domain can be represented as:

(2) $$L_{1}={\mathrm{an}}_{1}+\left(n_{1}-1\right)d_{1},$$

and

(3) $$L_{2}={\mathrm{bn}}_{2}+\left(n_{2}-1\right)d_{2}.$$

Therefore, based on Equations (2) and (3), the surface, S, given by Equation (1) can be expressed as:

(4) $$S=\left({\mathrm{an}}_{1}+\left(n_{1}-1\right)d_{1}\right)\left({\mathrm{bn}}_{2}+\left(n_{2}-1\right)d_{2}\right)$$

Again using Equations (2) and (3), the number of keratinocytes in both directions can be calculated as:

(5) $$n_{1}=\frac{L_{1}+d_{1}}{a+d_{1}},$$

(6) $$n_{2}=\frac{L_{2}+d_{2}}{b+d_{2}}.$$

Substituting Equations (5) and (6) into (4), one obtains:

(7) $$S=\left(a\frac{L_{1}+d_{1}}{a+d_{1}}+\left(\frac{L_{1}+d_{1}}{a+d_{1}}-1\right)d_{1}\right)\left(b\frac{L_{2}+d_{2}}{b+d_{2}}+\left(\frac{L_{2}+d_{2}}{b+d_{2}}-1\right)d_{2}\right)$$

The part of surface, S, only occupied by keratinocytes can be expressed as:

(8) $$S_{n}=\left({\mathrm{an}}_{1}\right)\left({\mathrm{bn}}_{2}\right),$$

and after substituting Equations (5) and (6) into (8), the S_n_ is:

(9) $$S_{n}=a\frac{L_{1}+d_{1}}{a+d_{1}}b\frac{L_{2}+d_{2}}{b+d_{2}}.$$

The ratio of the surface of the skin filled with lipid channels to the total considered surface can be calculated as:

(10) $$\varepsilon =\frac{S-S_{n}}{S}.$$

After the substitution of Equations (7) and (9) into (10), one obtains:

(11) $$\varepsilon =\frac{\left(a\frac{L_{1}+d_{1}}{a+d_{1}}+\left(\frac{L_{1}+d_{1}}{a+d_{1}}-1\right)d_{1}\right)\left(b\frac{L_{2}+d_{2}}{b+d_{2}}+\left(\frac{L_{2}+d_{2}}{b+d_{2}}-1\right)d_{2}\right)-a\frac{L_{1}+d_{1}}{a+d_{1}}b\frac{L_{2}+d_{2}}{b+d_{2}}}{\left(a\frac{L_{1}+d_{1}}{a+d_{1}}+\left(\frac{L_{1}+d_{1}}{a+d_{1}}-1\right)d_{1}\right)\left(b\frac{L_{2}+d_{2}}{b+d_{2}}+\left(\frac{L_{2}+d_{2}}{b+d_{2}}-1\right)d_{2}\right)}$$

When it is assumed that all dimensions in both directions are the same, it means L_1_ = L_2_, a = b, and d_1_ = d_2_, and then n_1_ will be equal to n_2_, and Equation (11) can be written as:

(12) $$\varepsilon =\frac{{\left(a\frac{L+d}{a+d}+\left(\frac{L+d}{a+d}-1\right)d\right)}^{2}-a^{2}{\left(\frac{L+d}{a+d}\right)}^{2}}{{\left(a\frac{L+d}{a+d}+\left(\frac{L+d}{a+d}-1\right)d\right)}^{2}}.$$

The present work considers the three-dimensional brick-and-mortar model of the stratum corneum. The advantage of such an approach is that it is possible to consider the surface of the skin where the active substance was placed and the size of the area of the migration. Therefore, it allows for predicting the amount of the substance which will be transported through the skin and the efficiency of such transport. However, from the point of view of numerical simulation, such geometry can be difficult to use. The size of the skin covered with the substance is usually measured in centimeters, and the size of lipid channels is in the range of nanometers. Such big disproportionality creates many numerical problems and can be a source of errors and the lack of convergence of the solution. Additionally, the time of calculations can be very long and not acceptable. To solve these problems, the following attempt is proposed.

The three-dimensional brick-and-mortar model of the stratum corneum can be considered as the granular bed consisting of bricks of the size a∙b∙h placed regularly in the space of L_1_∙L_2_∙H. Such a structure can be replaced by the capillary tube, as was introduced by the concept of Kozeny Carman [16,17,18,19,20]. This approach is based on Equation (13), adjusted to the present case:

(13) $$r_{h}=\frac{\varepsilon }{a_{p}\left(1-\varepsilon \right)},$$

where ε is the space filled by lipids between keratinocytes, as given by Equation (11), a_p_ is the specific surface of keratinocyte, and r_h_ is the hydraulic radius of the model capillary. 

According to this theory, the specific surface of the grain in the bed can be defined as:

(14) $$a_{p}=\frac{S_{p}}{V_{p}},$$

where S_p_ isthe surface area of the walls of a single keratinocyte, and V_p_ is the volume of the single keratinocyte

Since in the present work the keratinocytes are considered as the bricks, the S_p_ and V_p_ can be calculated accordingly:

(15) $$S_{p}=2\left(\mathrm{ab}+\mathrm{bh}+\mathrm{ah}\right),$$

and

(16) $$V_{p}=\mathrm{abh}.$$

After the substitution of Equations (14) and (15) into (13), the specific area is expressed as:

(17) $$a_{p}=\frac{\left(\mathrm{ab}+\mathrm{bh}+\mathrm{ah}\right)}{\mathrm{abh}}.$$

If the keratinocyte has a square base, it means a = b, then Equation (17) takes the form:

(18) $$a_{p}=\frac{\left(a^{2}+2\mathrm{ah}\right)}{a^{2}h}.$$

The hydraulic radius given by Equation (13) can be, after the substitution of Equations (11) and (18), calculated as follows:

(19) $$r_{h}=\frac{\frac{\left(a\frac{L_{1}+d_{1}}{a+d_{1}}+\left(\frac{L_{1}+d_{1}}{a+d_{1}}-1\right)d_{1}\right)\left(b\frac{L_{2}+d_{2}}{b+d_{2}}+\left(\frac{L_{2}+d_{2}}{b+d_{2}}-1\right)d_{2}\right)-a\frac{L_{1}+d_{1}}{a+d_{1}}b\frac{L_{2}+d_{2}}{b+d_{2}}}{\left(a\frac{L_{1}+d_{1}}{a+d_{1}}+\left(\frac{L_{1}+d_{1}}{a+d_{1}}-1\right)d_{1}\right)\left(b\frac{L_{2}+d_{2}}{b+d_{2}}+\left(\frac{L_{2}+d_{2}}{b+d_{2}}-1\right)d_{2}\right)}}{\frac{\left(\mathrm{ab}+\mathrm{bh}+\mathrm{ah}\right)}{\mathrm{abh}}\left(1-\frac{\left(a\frac{L_{1}+d_{1}}{a+d_{1}}+\left(\frac{L_{1}+d_{1}}{a+d_{1}}-1\right)d_{1}\right)\left(b\frac{L_{2}+d_{2}}{b+d_{2}}+\left(\frac{L_{2}+d_{2}}{b+d_{2}}-1\right)d_{2}\right)-a\frac{L_{1}+d_{1}}{a+d_{1}}b\frac{L_{2}+d_{2}}{b+d_{2}}}{\left(a\frac{L_{1}+d_{1}}{a+d_{1}}+\left(\frac{L_{1}+d_{1}}{a+d_{1}}-1\right)d_{1}\right)\left(b\frac{L_{2}+d_{2}}{b+d_{2}}+\left(\frac{L_{2}+d_{2}}{b+d_{2}}-1\right)d_{2}\right)}\right)}$$

For the case when L_1_ = L_2_, a = b, and d_1_ = d_2_, the hydraulic radius can be calculated using the formula:

(20) $$r_{h}=\frac{\frac{{\left(a\frac{L+d}{a+d}+\left(\frac{L+d}{a+d}-1\right)d\right)}^{2}-a^{2}{\left(\frac{L+d}{a+d}\right)}^{2}}{{\left(a\frac{L+d}{a+d}+\left(\frac{L+d}{a+d}-1\right)d\right)}^{2}}}{\frac{\left(a^{2}+2\mathrm{ah}\right)}{a^{2}h}({1-\frac{{\left(a\frac{L+d}{a+d}+\left(\frac{L+d}{a+d}-1\right)d\right)}^{2}-a^{2}{\left(\frac{L+d}{a+d}\right)}^{2}}{{\left(a\frac{L+d}{a+d}+\left(\frac{L+d}{a+d}-1\right)d\right)}^{2}}}^{L})}$$

The assumed approach allows us to consider the diffusion process, not in the brick-and-mortar complicated structure with a big difference between the size of the domain, measured in millimeters and centimeters with nanochannels, but by the model where the complicated layout of lipid channels is replaced by the single tube of radius r_h_. The length of such a tube can be considered equal to the thickness, h, of a single keratinocyte layer. Such an approach allows also for easier modeling of diffusion phenomena.

### 2.2. Model of a Diffusion Process of Nanoparticles

Transport of the nanoparticles along the narrow channel—the capillary—can be described using Fick’s first law:

(21) $$J=D_{\mathrm{eff}}\frac{\mathrm{dc}}{\mathrm{dx}},$$

where J is the mass flow rate of the migrating component (kg/m^2^s), D_eff_ is the effective diffusion coefficient (m^2^/s), c is the concentration of the diffusing component (kg/m^3^), and x is the diffusion distance (m).

The mass flow rate of the diffusing stream can be expressed using the following equation:

(22) $$J=\frac{m_{p}}{\mathrm{At}},$$

where m_p_ is the mass of the transferred component (kg), A is the surface area of the diffusion (m^2^), and t is the diffusion time (s).

After the substitution of Equation (22) into (21), one obtains:

(23) $$\frac{m_{p}}{\mathrm{At}}=D_{\mathrm{eff}}\frac{\mathrm{dc}}{\mathrm{dx}}.$$

Considering the transport of nanoparticles, their transferred mass, m_p_, is:

(24) $$m_{p}=N_{\mathrm{pt}}\rho_{p}V_{p},$$

where N_pt_ is the number of diffusing particles (−), ρ_p_ is the density of nanoparticles (kg/m^3^), and V_p_ is the volume of the single nanoparticle (m^3^).

The presented research considers the movement of nanoparticles in the narrow channels of the stratum corneum replaced by the narrow capillary as the result of the modeling assumptions. Since the dimensions of the diffusing elements can vary from a single to a dozen nanometers or so, their movement can be treated as Brownian movement resulting from the thermal action of molecules in the lipid layer. Considering such a phenomenon, Equation (23) can be presented as follows:

(25) $$\frac{N_{\mathrm{pt}}\rho_{p}V_{p}}{\mathrm{At}}=D_{\mathrm{eff}}\frac{\frac{N_{p0}\rho_{p}V_{p}-N_{\mathrm{pt}}\rho_{p}V_{p}}{V}}{h},$$

and after simplification:

(26) $$\frac{N_{\mathrm{pt}}}{\mathrm{At}}=D_{\mathrm{eff}}\frac{\frac{N_{p0}}{V}-\frac{N_{\mathrm{pt}}}{V}}{h},$$

where N_p0_ is the initial number of particles in the channel (−), N_pt_ is the number of particles which diffused in time t—the number of particles that passed until the end of the channel in time t (−), N_p0_/V is the initial concentration of particles related to the size of the diffusion volume in the system (1/m^3^), N_pt_/V is the concentration of particles that passed the volume until the end and related to the volume of the system t (1/m^3^), and h is the length of the channel (m).

After further rearrangements, Equation (26) can be written as:

(27) $$\frac{N_{\mathrm{pt}}}{\mathrm{At}}=D_{\mathrm{eff}}\frac{N_{p0}-N_{\mathrm{pt}}}{\mathrm{Vh}}.$$

Volume, V, where the diffusion process occurs can be expressed as:

(28) V=Ah.

After the substitution of this equation to (26) and simplification, one can obtain:

(29) $$\frac{N_{\mathrm{pt}}}{t}=D_{\mathrm{eff}}\frac{N_{p0}-N_{\mathrm{pt}}}{h^{2}}.$$

Further rearrangements lead to:

(30) $$N_{\mathrm{pt}}h^{2}=D_{\mathrm{eff}}\left(N_{p0}-N_{\mathrm{pt}}\right)t,$$

(31) $$N_{\mathrm{pt}}h^{2}=D_{\mathrm{eff}}N_{p0}t-D_{\mathrm{eff}}N_{\mathrm{pt}}t,$$

(32) $$N_{\mathrm{pt}}h^{2}+D_{\mathrm{eff}}N_{\mathrm{pt}}t=D_{\mathrm{eff}}N_{p0}t,$$

(33) $$N_{\mathrm{pt}}\left(h^{2}+D_{\mathrm{eff}}t\right)=D_{\mathrm{eff}}N_{p0}t,$$

(34) $$\frac{N_{\mathrm{pt}}}{N_{p0}}=\frac{D_{\mathrm{eff}}t}{\left(h^{2}+D_{\mathrm{eff}}t\right)}.$$

Equation (34) is known in the literature, in its mathematical form, as Hill’s equation [11]. This formula, with a different meaning of variables and parameters, is very often used to approximate the processes of adsorption, the kinetics of reactions, and so on, when, for instance, the product concentration or the number of particles approaches the final, maximal, or minimal value. It is very often presented with the addition of the n parameter, which yields the following expression:

(35) $$\frac{N_{\mathrm{pt}}}{N_{p0}}=\frac{D_{\mathrm{eff}}t^{n}}{\left(h^{2}+D_{\mathrm{eff}}t^{n}\right)}.$$

## 3. Numerical Simulation of the Diffusion Processes

The numerical simulations were performed using the COMSOL Multiphysics v.6 [14,15] programs package. The numerical domain was constructed as the narrow capillary as it results from the modeling considerations described in Section 2.1. The diameter of the duct was calculated using Equation (20). It was assumed that the surface of the stratum corneum, which is the donor of the substance migrating into the skin, has the size L_1_ = L_2_ = 0.02 m (2 cm). It was also assumed that the size of the keratinocytes is equal to a = b = 2·10^−5^ m (20 μm). The width of the gaps between keratinocytes was assumed as d_1_ = d_2_ = 5·10^−8^ m (50 nm). The thickness of a single layer, h, was equal to 1·10^−5^ m (1 μm). Such dimensions of elements of the stratum corneum give the hydraulic radius of the capillary, calculated using Equation (20), equal to r_h_ = 12.5 nm. 

Therefore, the numerical calculations were performed in the three-dimensional domain of the diameter of d_c_ = 25 nm and the height of h = 1 μm. Such length represents the thickness of the single layer of keratinocytes and is responsible for the resistance to diffusion. Compared to this, the resistance of lipid horizontal layers between keratinocytes is negligible. 

**Figure 3:**
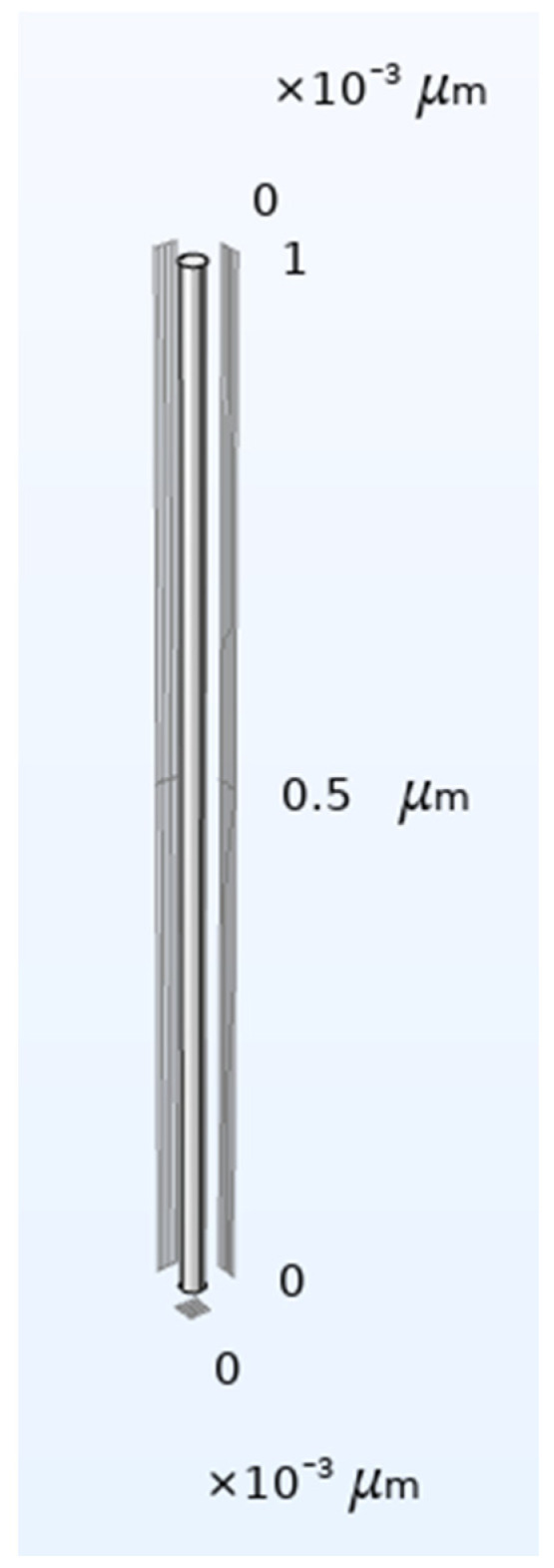
Geometry of the numerical domain.

The numerical domain resulting from the above dimensions is presented in Figure 3.

The diameter, d_p_, of the migrating particles was 1, 5, 10, and 15 nm. It means that it was comparable with the capillary diameter, d_c_, and this ratio was equal to, respectively, 0.2, 0.4, 0.6, and 0.75. Their density was 2200 kg/m^3^. Such a density is typical for most mineral powders, such as silica or others. The density of the liquid in which the diffusion occurred was assumed to be 1000 kg/m^3^. The viscosity of this liquid was equal to 10, 20, and 50 mPa·s.

At the assumed size of the particles, their movement in the liquid was treated as the Brownian motion, and such a model of diffusion was applied. In this motion, the acting forces and the moving particle are counterbalanced by Stokes’ drag force, resulting from the viscosity of the surrounding liquid. These forces were calculated, according to the COMSOL programs’ manuals [12], from the following formulae:

Brownian force:

(36) $$F_{B}=\zeta \sqrt{\frac{6{\pi k}_{B}{\eta \mathrm{Td}}_{p}}{\Delta t}},$$

Stokes’ force:

(37) $$F_{S}=\frac{18{\eta m}_{p}v}{\rho_{p}d_{}^{2}}.$$

where m_p_ is the mass of the particle (kg), v is the velocity of the particle (m/s), η is the liquid viscosity (Pa∙s), ρ_p_ is the particle density (kg/m^3^), d is the particle diameter (m), k_B_ is the Boltzman constant (J/K), T is the temperature (K), Δt is the time step (s), and ζ is a random number (−).

The density of the mesh was defined after the preliminary calculation. Its final density was such that further increases did not influence the results. Additionally, the number of particles in the system was evaluated on an experimental basis. The value of 5000 was big enough that a further increase did not change the final solutions. The particles in the simulation process were released at 30 nm from the upper surface of the domain. 

Two types of possible contacts of the particles with the wall of the tube were assumed: bouncing contact with full reflection and full preservation of energy of the particle on the upper and side walls of the domain, and freezing contact, lack of reflection, and total loss of energy on the bottom surface of the domain. Such an approach allowed us to study the efficiency of the diffusion process.

## 4. Results of the Numerical Experiments and Discussion

**Figure 4:**
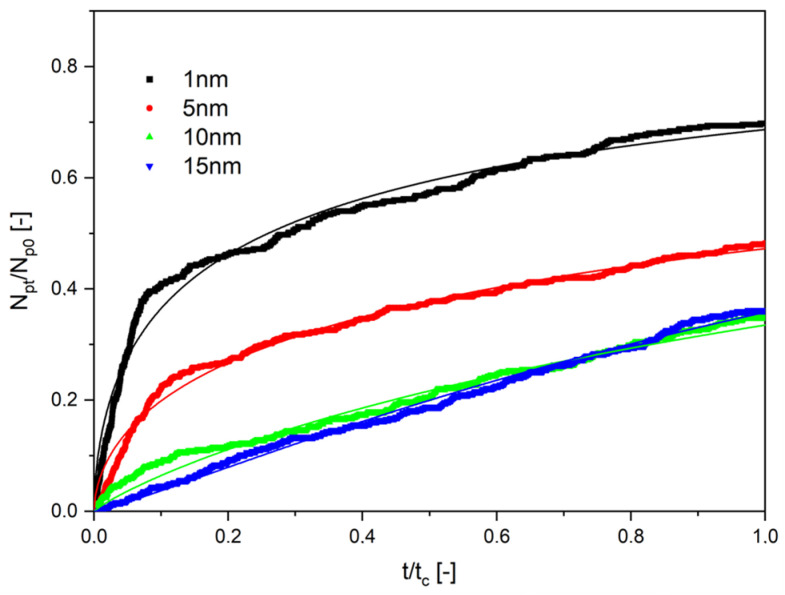
The dependence on the normalized number of particles as a function of normalized time. The viscosity of liquid was 10 mPa∙s for particles of different diameters.

**Figure 5:**
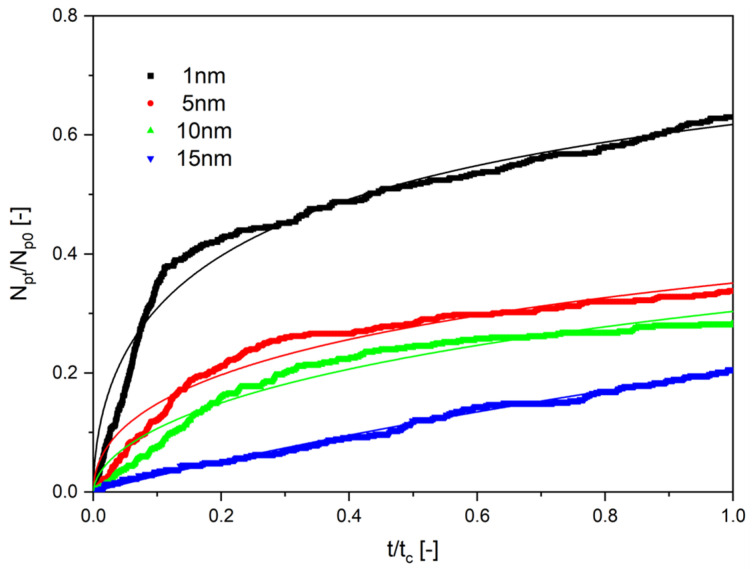
The dependence on the normalized number of particles as a function of normalized time. The viscosity of the liquid was 20 mPa∙s for particles of different diameters.

**Figure 6:**
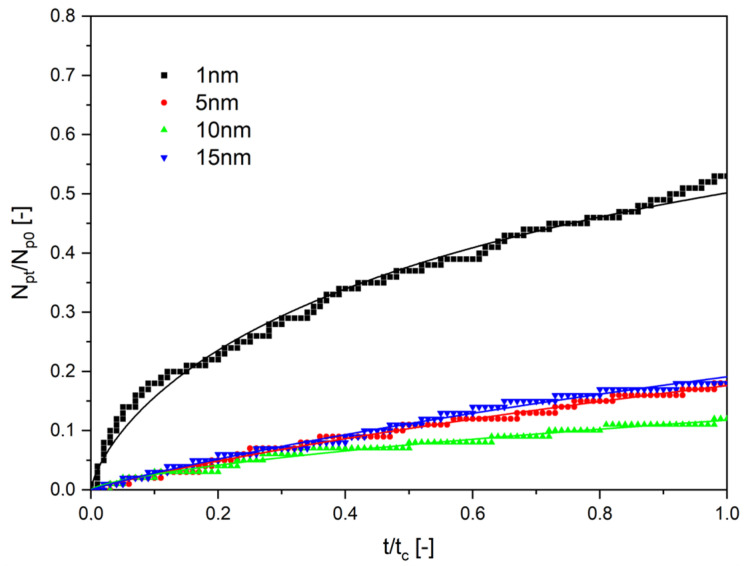
The dependence on the normalized number of particles as a function of normalized time. The viscosity of the liquid was 50 mPa∙s for particles of different diameters.

Results of the numerical investigations are presented in Figure 4, Figure 5 and Figure 6. They are presented as the ratio of the number of particles that diffused through the channel, N_p_, to its end to the initial number of particles starting the diffusion, N_p0_, as a function of the normalized time. The normalized time was the ratio of the given time of calculations, t, to the final time of computations, t_c_. In all cases, the end time was equal to 500 s. Thin lines in Figure 4, Figure 5 and Figure 6 are the results of the approximation of data by Equation (35), obtained using the Microcal Origin Pro program.

**Figure 7:**
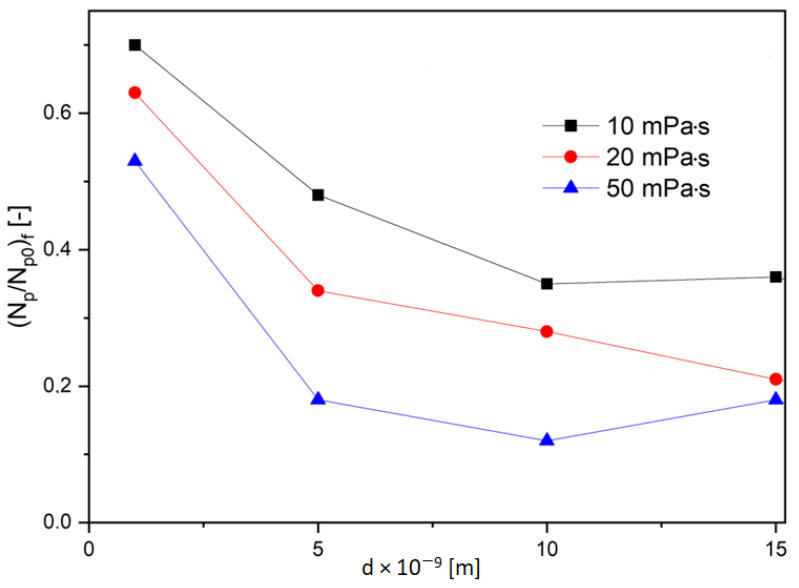
The dependence of the number of particles as a function of their diameter for liquids of different viscosities.

It results from these graphs that the number of particles that diffused through the capillary at the time of calculations is dependent on their size and the viscosity of the liquid in which the diffusion occurs. These dependencies are shown in Figure 7. One can see that the rising viscosity of the liquid and the rising size of the particles decreased the number of particles diffusing through the capillary. Therefore, the amount of the substance decreased with time, and the velocity of the diffusion of the same amount was longer. 

The results presented in Figure 4, Figure 5 and Figure 6 have been approximated using Equation (35). The results are drawn as continuous lines on the graphs and the numerical values of the parameters D_eff_ and n are shown in Table 1. The length of diffusion, h, was assumed to be equal to 10^−6^ m. It results from the numerical experiments that the effective diffusion coefficients change in the range from 2.36·10^−13^ to 21.9·10^−12^ m^2^/s. 

Generally, the literature concerning experimental investigations of the transport of nanoparticles through the skin layers is quite extensive. However, the studies are mainly focused on the point: will nanoparticles permeate through the epidermis or not? Results are very different because the definition of the size of nanoparticles is still not exact. One can find results concerning the transport of nanoparticles of the size of several hundreds of nanometers and particles smaller than twenty nanometers. It results from [10,21] that only such small particles can permeate the stratum corneum. 

Therefore, based on the above, the experimental validation of the results of this paper can be performed for the migration of such small particles. Here, the extensive experimental study of the diffusion of silver nanoparticles of different shapes, through the mouse skin, has been presented [22]. It was found that the value of the diffusion coefficient of nanoparticles of different shapes in the investigated experimental material was in the range of 4.3·10^−13^ to 4.9·10^−13^ m^2^/s for spherical ones of the size of 5 nm. Slightly different values were obtained for other shapes. 

The diffusion of gold nanoparticles through the skin was investigated by the authors of [23]. It was found that for the range of sizes of from two to five nanometers, one can expect the values of the diffusion coefficients to range from 0.5·10^−11^ to 2·10^−11^ m^2^/s. 

The transport of big molecules was the subject of the research presented in [24]. The authors have determined that diffusion coefficients of dextran were in the range of 8·10^−12^ to 20·10^−12^ m^2^/s. 

The papers which deal with the transport in channels of micrometric diameter [8] or chemical compounds through the stratum corneum report values of diffusion coefficients in the range from 10^−13^ to 10^−12^ m^2^/s [25]. 

It results from Table 1 that the values of the effective diffusion coefficients decreased with the growing diameter of nanoparticles and with the increase of the viscosity, in which the transport occurs. The values of the determination coefficient, R^2^, showed good agreement between data and approximations obtained using Equation (35).

The values of D_eff_ from Table 1 can be compared with the diffusion coefficient of particles in a liquid, as predicted by the Stokes-Einstein equation in the form:

(38) $$D=\frac{k_{b}T}{6{\pi \eta d}_{p}}$$

where k_B_ = 1.381·10^−23^ (J/K) is the Boltzmann constant, T = 298 (K) is the temperature,

η is the fluid viscosity (mPa∙s), and d_p_ is the particle diameter (m).

The results of the calculations, together with the ratio D/D_eff_, are presented in Table 2.

It results from the performed calculation (see Table 2) that the values of the effective diffusion coefficient, D_eff_, were generally a few times smaller than the values of D. Such a finding allows to estimate the restricted role of the walls of the capillaries and in the real stratum corneum on the velocity of diffusion processes.

## 5. Conclusions

The presented considerations showed two essential approaches to the modeling of diffusion of the nanoparticles through the most resistive layer of skin—the stratum corneum. 

The first model is related to the geometry of the skin. It represents the conversion of the three-dimensional structure in the single capillary. Such an approach provides a much more treatable image of the penetration routes of the particles in the skin. Additionally, if numerical simulations are applied, such a geometry is much easier for building the numerical model. The stratum corneum consists of keratinocytes of a size of several dozens of micrometers separated by the narrow channels of a nanometric width. Due to the disproportionality between these elements, numerical calculation can be very time-consuming, with very small progress in the convergence of solutions. 

This three-dimensional model also gives the possibility of including in the predictions the surface of the skin, on which a substance was placed and migrated into the organism. 

The second model deals directly with the diffusion process. Taking the advantage of the first one, it allows to easily define the distance of the diffusion as the length of the capillary. Additionally, the derived equation allows to predict the number of migrated components as a function of the time and the distance. 

The applicability of the geometrical model and the model of diffusion allowed us to describe with acceptable accuracy the results of numerical simulations. The parameters of the diffusion model estimated based on the approximation of the numerical data yield values of the effective diffusion coefficient. These values are in the range of data available for diffusion coefficients of nanoparticles through the stratum corneum. 

## Figures and Tables

**Table 1:** Values of the effective diffusion coefficient, D_eff_, and the n parameter.

d∙10^9^ m	D_eff_ ·10^13^ (m^2^/s)			n (−)		
	η 10 mPa∙s	η 20 mPa∙s	η 50 mPa∙s	η 10 mPa∙s	η 20 mPa∙s	η 50 mPa∙s
1 (R^2^)	21.9 (0.987)	16.2 (0.990)	10.1 (0.989)	0.581	0.557	0.734
5 (R^2^)	8.96 (0.991)	5.41 (0.992)	2.14 (0.983)	0.557	0.491	0.889
10 (R^2^)	5.54 (0.982)	4.36 (0.989	1.33 (0.984)	0.863	0.561	0.697
15 (R^2^)	5.04 (0.993)	2.54 (0.991)	2.36 (0.985)	1.159	0.958	0.907

**Table 2:** Comparison of values of D calculated from Equation (38) with D_eff_ values from Table 1.

d∙10^9^ m	η = 10 mPa∙s		η = 20 mPa∙s		η = 50 mPa∙s	
	D 10^13^ (m^2^/s)	D/D_eff_	D 10^13^ (m^2^/s)	D/D_eff_	D 10^13^ (m^2^/s)	D/D_eff_
1	21.5	9.79	107	6.62	42.9	4.24
5	4.29	4.79	21.5	3.96	8.58	4.01
10	2.15	4.25	10.7	2.46	4.29	3.22
15	1.43	2.56	7.15	2.81	2.86	1.21

## Data Availability

Not applicable.
